# Primary intracranial malignant melanomas: A case series with literature review

**DOI:** 10.1097/MD.0000000000040334

**Published:** 2024-11-01

**Authors:** Lifeng Chen, Yang Yang, Dongmei Li, Bo Bu

**Affiliations:** a Department of Neurosurgery, The First Medical Center of the Chinese PLA General Hospital, Beijing, China; b Department of Neurology, The Second Medical Center of the Chinese PLA General Hospital, Beijing, China.

**Keywords:** case report, central nervous system, diagnosis, primary malignant melanoma, treatment

## Abstract

**Rationale:**

There is a high chance of misdiagnosis and limited knowledge regarding therapeutic strategies owing to the rarity of primary intracranial malignant melanoma (PIMM). The objective of the present study was to evaluate the clinical features, treatment modalities, and outcomes of patients with histologically proven PIMM.

**Patient concerns:**

Data of 15 patients with PIMM admitted to the Chinese People’s Liberation Army General Hospital in a 14-year period between January 2005 and January 2019 were collected. Clinical presentations, pathology, surgical strategies, adjuvant treatment, and prognosis were retrospectively analyzed.

**Diagnoses:**

CT showed iso- or high-density lesions in 12 cases (80%). MRI revealed short T1 and slightly short T2 in 14 cases (93.3%).The tumors showed mild or no enhancement on enhanced MRI. The patients were eventually diagnosed with PIMM through pathological examination.

**Interventions:**

The treatment modalities included radical resection followed by conventional radiotherapy (RT, n = 12) and subtotal resection followed by stereotactic radiosurgery (n = 3).

**Outcomes:**

All 15 patients had either recurrence or metastasis at an average of 14.7 months (range, 6–23 months) after surgery. In total, 14 patients (93.3%) succumbed to disease, with a mean overall survival of 22 months (range, 6–36 months). The median survival time was 23 months. The overall survival rates at 1, 2, and 3 years were 80, 47, and 13%, respectively. Radical resection with RT was associated with longer overall survival (log-rank, *P* < .05) than subtotal resection followed by stereotactic radiosurgery.

**Lessons:**

PIMM is an extremely rare tumor with a poor prognosis. Radical resection with RT may result in a longer overall survival rate. Targeted immunotherapy may be a promising treatment option for PIMM.

## 1. Introduction

Primary intracranial malignant melanoma (PIMM) is a rare malignancy, representing 0.07% of all cases of intracranial tumors.^[1]^ In addition, it only accounts for ~1% of all melanoma cases.^[2]^ According to their pathological behavior, PIMMs are classified into 2 types: the solitary type presents as a nodular mass, and the diffuse type infiltrates the pia mater and involves the subarachnoid space.^[3]^ Due to the rarity of PIMMs and the lack of randomized studies, there is a high chance of misdiagnosis and limited knowledge regarding the type of therapeutic strategies effective against this disease.^[1–6]^ Although the preoperative symptoms and imaging findings are similar to those of meningiomas or benign melanocytomas, PIMM has a variable prognosis. It is important to accurately diagnose and identify the exact extent of the tumor area.^[5]^ Resection or biopsy combined with adjuvant treatment, including whole-brain RT (WBRT), stereotactic radiosurgery (SRS), chemotherapy, and/or immunotherapy, have all been recommended.^[3]^ However, gross total resection has been reported to improve the outcomes of PIMMs.^[3]^ The benefits of radiation therapy, chemotherapy, and immunotherapy for PIMM remain controversial, and the prognosis of this disease remains poor.^[1–6]^ Therefore, in the present study, the clinical features, treatment modalities, and outcomes of patients with histologically proven PIMM were retrospectively reviewed and discussed.

## 2. Materials and methods

### Patients

During a 14-year period from January 2005 to January 2019, there were 15 consecutive patients with pathologically confirmed PIMM treated in the Department of Neurosurgery of the Chinese People’s Liberation Army General Hospital, including 11 men and 4 women, with a mean age 37.9 years (19–61 years).

This study was approved by the Ethics Board of the Chinese People’s Liberation Army General Hospital. Patients with a postoperative pathological diagnosis of malignant melanoma were included based on the following criteria outlined by Willis^[4]^: no skin or eye melanoma, no history of skin or eye melanoma surgery, and no extracranial visceral melanoma metastases. Clinical information, including the clinical presentation, imaging, and treatments performed, was retrospectively collected and analyzed from medical records, clinical notes, and surgical reports. Follow-up information at 3 months postoperatively and at 1-year intervals after surgery was obtained through outpatient visits or telephone interviews.

### Patient evaluation

CT was performed in 12 patients. MRI with T1, T2, and T1 enhancements was performed in all 15 patients before and after surgical treatment. Tumor size was defined as the largest tumor diameter on MRI. Gross total resection (GTR) was defined as 100% gross resection of the tumor. There was no residual tumor on MRI within 3 months of the operation. Subtotal resection (STR) was defined as the removal of <100% but >90% of the tumor. Any postoperative MRI revealing tumors that increased in size were considered tumor recurrence or tumor regrowth. All 15 patients were followed-up using neuroimaging and neurological examinations. Neurological and imaging examinations were performed preoperatively, 3 months postoperatively, and at 1-year intervals after surgery. The Karnofsky Performance Status score^[7]^ was also determined for each patient based on the clinical evaluation.

Surgical tumor specimens were fixed in formaldehyde and embedded in paraffin. They were then subjected to microscopic and immunohistochemical analyses. In total, 2 senior neuropathologists independently verified the histological examinations.

### Statistical analysis

The characteristics of the patients were described using descriptive statistics for N (%). Overall survival, progression-free survival, and efficacy of treatment modalities were analyzed using the Kaplan–Meier method. Univariate comparisons of parameters were performed using log-rank analysis. *P* < .05 was considered to indicate a statistically significant. All statistical analyses were performed using SPSS, version 19.0 (IBM Corp.).

## 3. Results

### Preoperative characteristics

**Figure 1.**
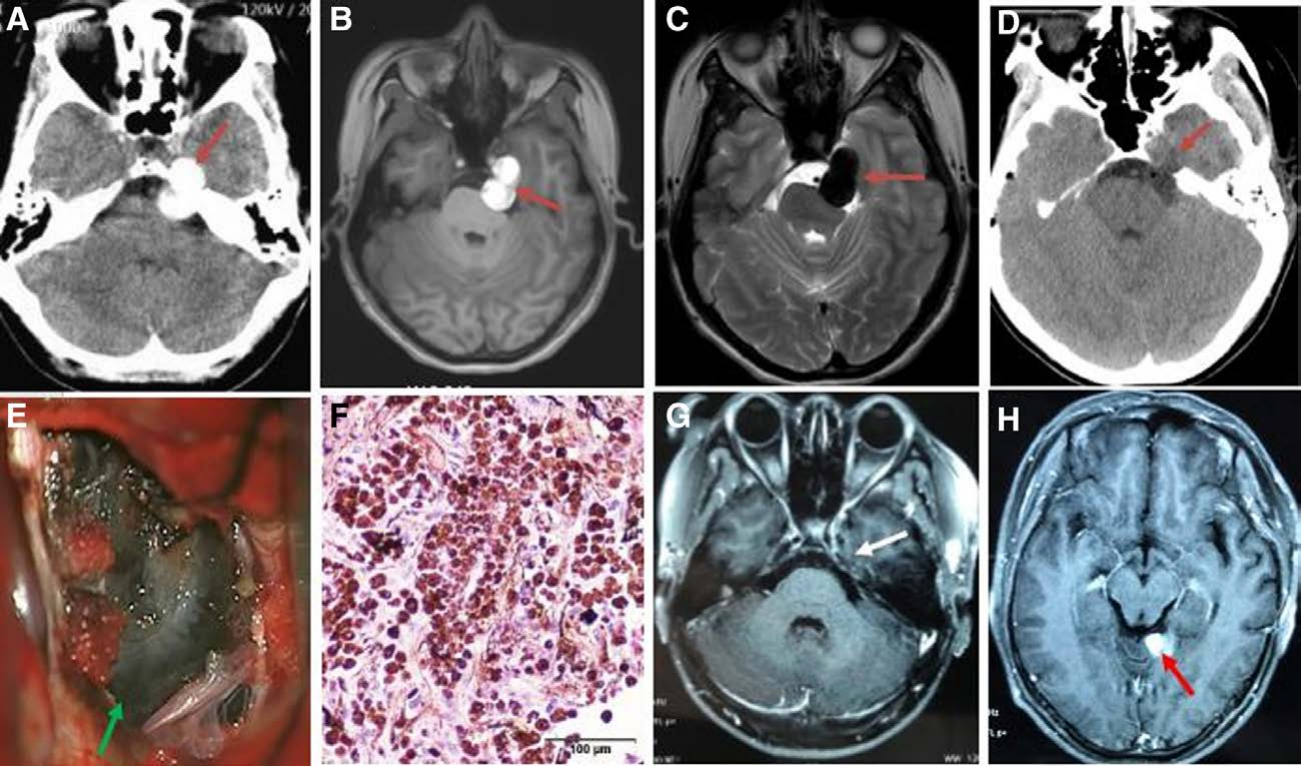
Preoperative, intraoperative and postoperative imaging with pathological examination of a 29-year-old female patient with left petrous apex melanoma. (A) Preoperative axial CT showed a high-density lesion (red arrow). (B) Preoperative axial T1WI showed an irregular, high-signal lobulated lesion (red arrow). (C) Preoperative axial T2WI showed an irregular, low-signal lobulated lesion (red arrow). (D) Axial CT at 1 day after surgery showed total tumor resection (red arrow). (E) Intraoperative imaging showed a melanoma (green arrow) with clear boundary. (F) Postoperative H&E staining at ×100 magnification showed the tumor tissue to be rich in melanin. (G) Axial T1-weighted contrast-enhanced MRI at 12 months after surgery showed total tumor resection (white arrow). (H) Axial T1-weighted contrast-enhanced MRI at 22 months after surgery showed melanoma metastasis (red arrow) of tentorial. WI = weighted image.

**Figure 2.**
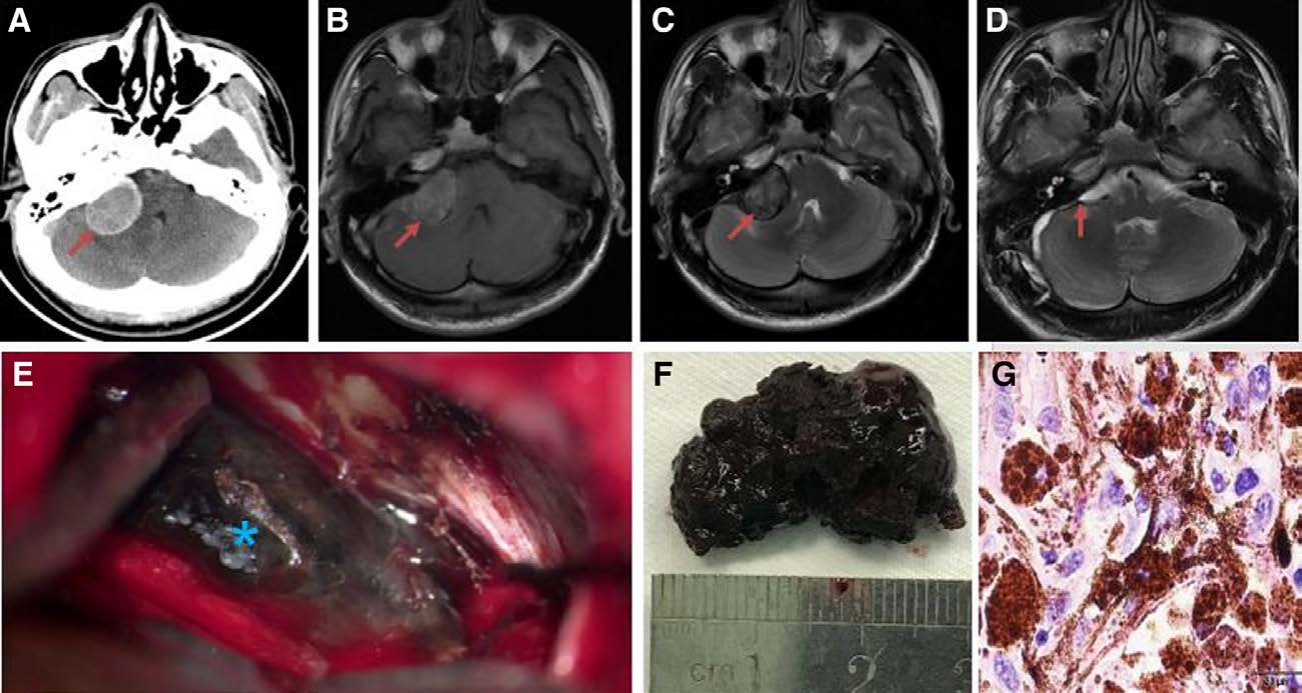
Preoperative and postoperative imaging, specimens and pathological examination of a 28-year-old male patient with right cerebellopontine angle melanoma. (A) Preoperative axial CT showed a high-density round lesion (red arrow). (B) Preoperative axial T1WI showed a high-signal round lesion (red arrow). (C) Preoperative axial T2WI showed a low-signal round lesion (red arrow). (D) Postoperative axial T2WI showed total tumor resection (red arrow). (E) Intraoperative imaging showed a black melanoma (asterisk). (F) Melanoma specimen. (G) Postoperative H&E staining at ×400 showed heteromorphous large cells with obvious nucleoli. WI = weighted image.

**Figure 3.**
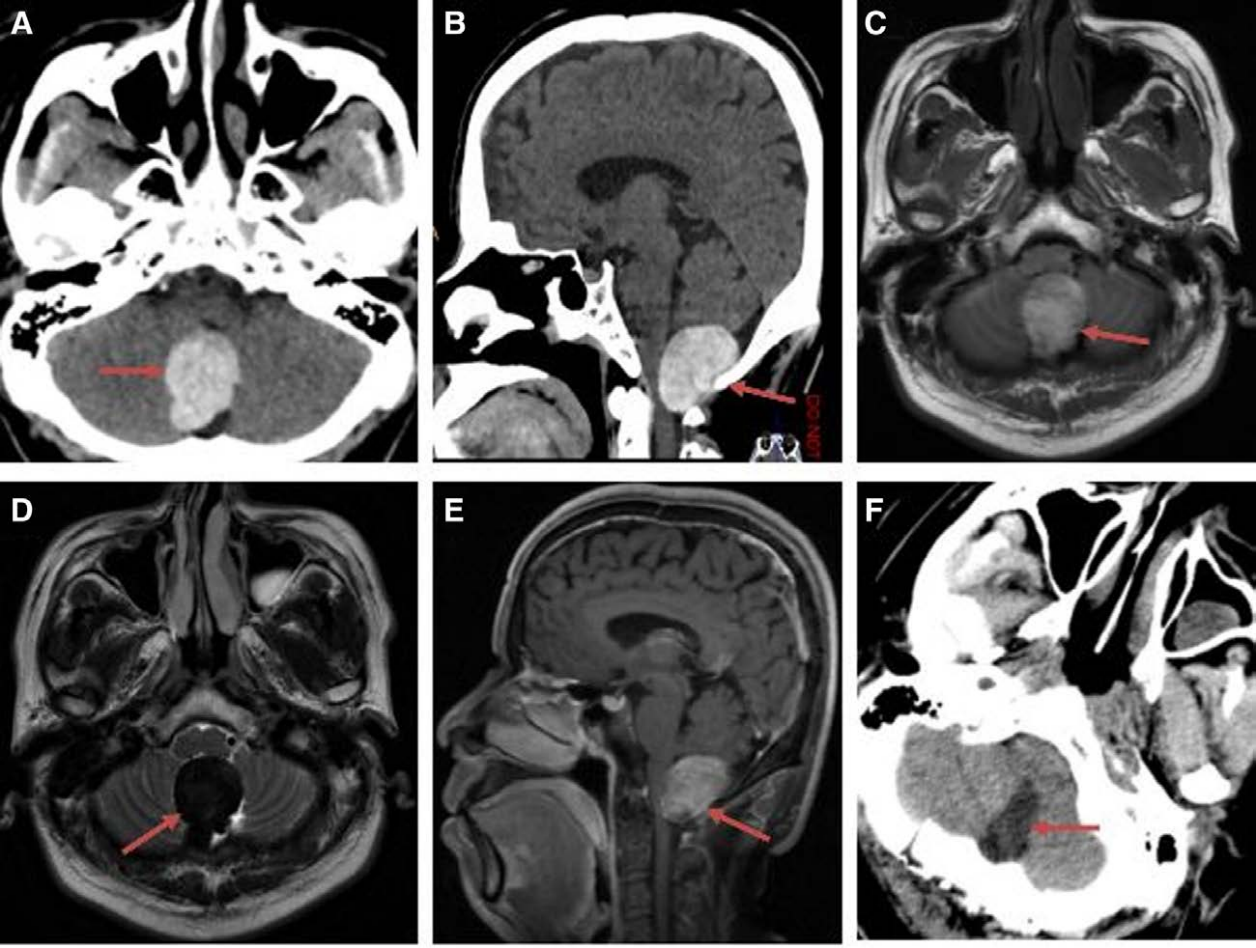
Preoperative and postoperative imaging of a 43-year-old male patient with foramen magnum melanoma. (A) Preoperative axial CT showed a high-density round lesion (red arrow). (B) Preoperative sagittal CT showed a high-density round lesion (red arrow). (C) Preoperative axial T1WI showed a high-signal round lesion (red arrow). (D) Preoperative axial T2WI showed a low-signal round lesion (red arrow). (E) Preoperative sagittal T1-weighted contrast-enhanced MRI showed a mild enhancement lesion (red arrow). (F) Axial CT at 1 day after surgery showed total tumor resection (red arrow). WI = weighted image.

**Figure 4.**
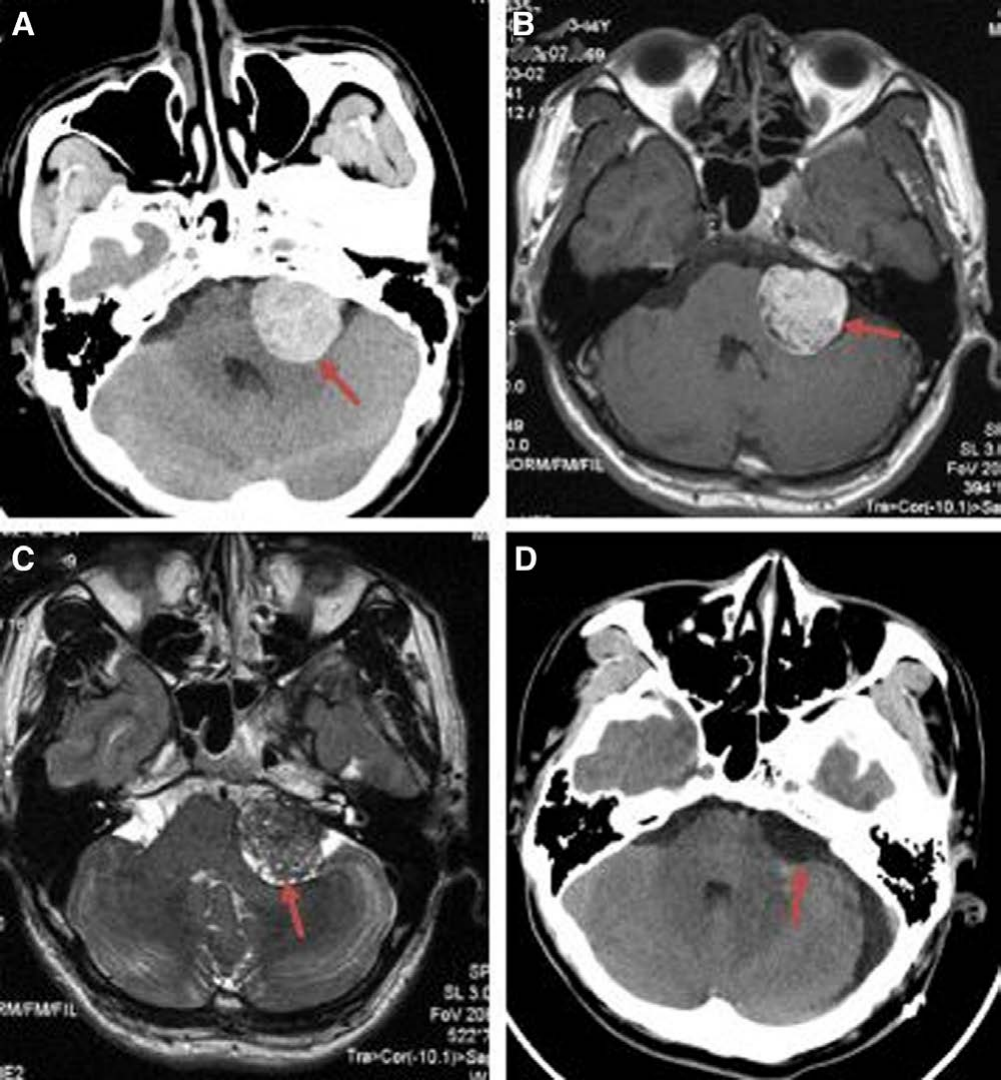
Preoperative and postoperative imaging of a 34-year-old male patient with left cerebellopontine angle melanoma. (A) Preoperative axial CT showed a high-density round lesion (red arrow). (B) Preoperative axial T1WI showed a high-signal round lesion (red arrow). (C) Preoperative axial T2WI showed a low-signal round lesion (red arrow). (D) Axial CT at 1 day after surgery showed total tumor resection (red arrow). WI = weighted image.

The most common symptom was headache (67%). The duration of the preoperative symptoms ranged from 6 days to 2 years, with an average of 6.3 months. The demographics and symptoms of the 15 patients are described in detail in Table 1. In total, 12 CT and 15 MRI scans were performed. CT showed iso- or high-density lesions in 12 cases (80%; Figs. 1A, 2A, 3A, and 4A). MRI revealed short T1 and slightly short T2 in 14 cases (93.3%; Figs. 1B, C, 2B, C, 3C, D, 4B, and C). The tumors showed mild or no enhancement on enhanced MRI (Fig. 3E). The maximum tumor was 2.5 to 8.0 cm, with an average of 4.6 cm. In addition, 14 patients had solitary tumors (93.3%), whereas 1 patient had 2 tumors located in the supratentorial region. Peritumoral brain edema was observed in 3 cases (20%). Based on the preoperative MRI, 3 patients (20%) were accurately diagnosed with melanoma. However, 12 patients were misdiagnosed with glioma, metastasis, schwannoma, or meningioma.

**Table 1:** Summary of 15 patient’s characteristics with primary malignant intracranial melanomas.

Characteristics	n = 15
Demographics	
Mean age, years	37.9 (range 19–61)
Males:females	11:4
Major symptoms and signs	
Headache	10 (67%)
Vomit	2 (13%)
Weakness of left limb	1 (7%)
Nystagmus	1 (7%)
Epilepsy	1 (7%)
Tinnitus and deafness	1 (7%)
Numbness of left face	1 (7%)
Facial paralysis	1 (7%)
Mean symptom duration, months	6.3 (range 6 days–2 years)
Tumor location	
Cerebral	9 (60%)
CPA	3 (20%)
Cerebellum	1 (7%)
Foramen magnum	1 (7%)
Middle fossa	1 (7%)
Mean size, cm	4.6 (range 2.5–8.0)
Median preoperative KPS	80 (range70–90)

KPS = Karnofsky Performance Status.

### Surgical characteristics

Tumors were removed using a variety of approaches according to their location. During the operation, the dura mater or the surrounding brain tissue was found to be black in 6 cases, with the tumor also being black (Figs. 1E, 2E, and F) in 14 cases and tan appearance in 1 case. The tumors were generally soft, with mild or moderate vascularization(Figs. 1E and 2E). In total, 2 cases with supratentorial tumors had hemorrhage. Intraoperatively, the section of the dura containing the tumors was either coagulated or removed. GTR was achieved in 12 patients (80%) (Figs. 1D, G, 2D, 3F, and 4D). STR was achieved in 3 cases (20%) because of their close relationship with the pyramidal tract.

Perioperative mortality was not observed. Facial paralysis was observed in 1 patient but recovered after 3 months. In addition, 1 patient had a postoperative intracranial infection. He was successfully treated with continuous drainage of the cerebrospinal fluid for 5 days. At the time of discharge, preoperative neurological function was either maintained or improved in 14 patients (93.3 %). The Karnofsky Performance Status scores ranged from 70 to 100 (Table 2).

**Table 2:** Outcome of treatment in15 patients with primary malignant intracranial melanomas.

Outcome	Total
Grade of resection	
Total resection	12
Subtotal resection	3
Recurrence after first treatment	
local recurrence alone	8 (53%)
Metastasis alone	5 (33%)
Both	2 (13%)
Location of metastasis	
Cerebellar	2 (40%)
Occipital lobe	2 (40%)
Multiple cerebral metastases	1 (20%)
Time to local recurrence (n = 10) Median, months	12 (range, 6–20)
Time to metastasis (n = 7) Median, months to extraneuralmetastasis (n = 5) Mean, months	18 (range, 12–23)
Time to overall death (n = 14)	
Median, months	23 (range, 6–36)
Mean follow-up duration, months	22.6 (range, 6–36)
Median postoperative KPS	90 (70–100)

KPS = Karnofsky Performance Status.

### Postoperative course and follow-up

**Figure 5.**
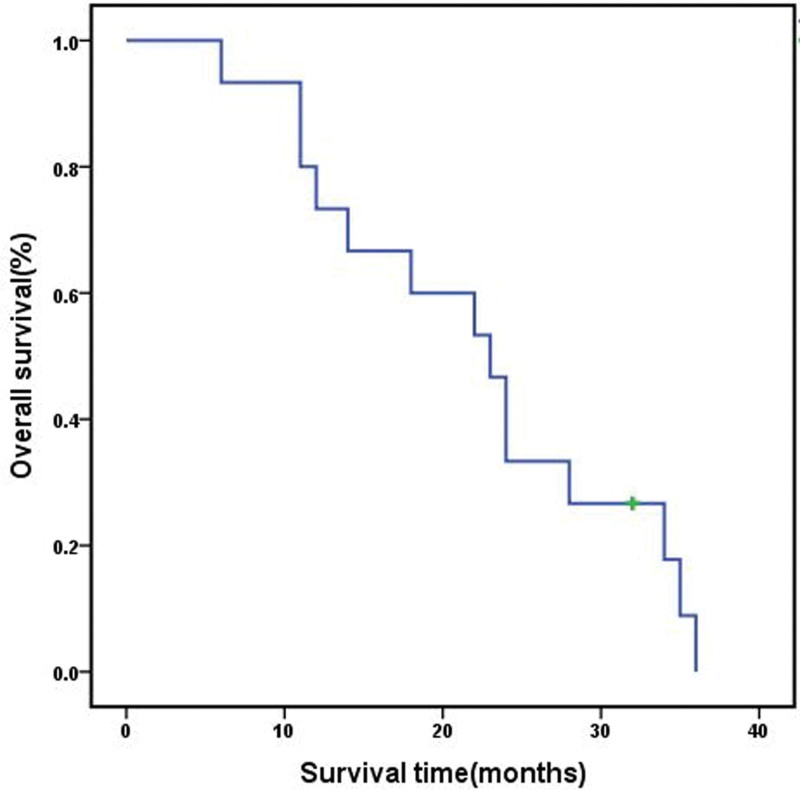
Kaplan–Meier curve showing the overall survival of the 15 patients.

**Figure 6.**
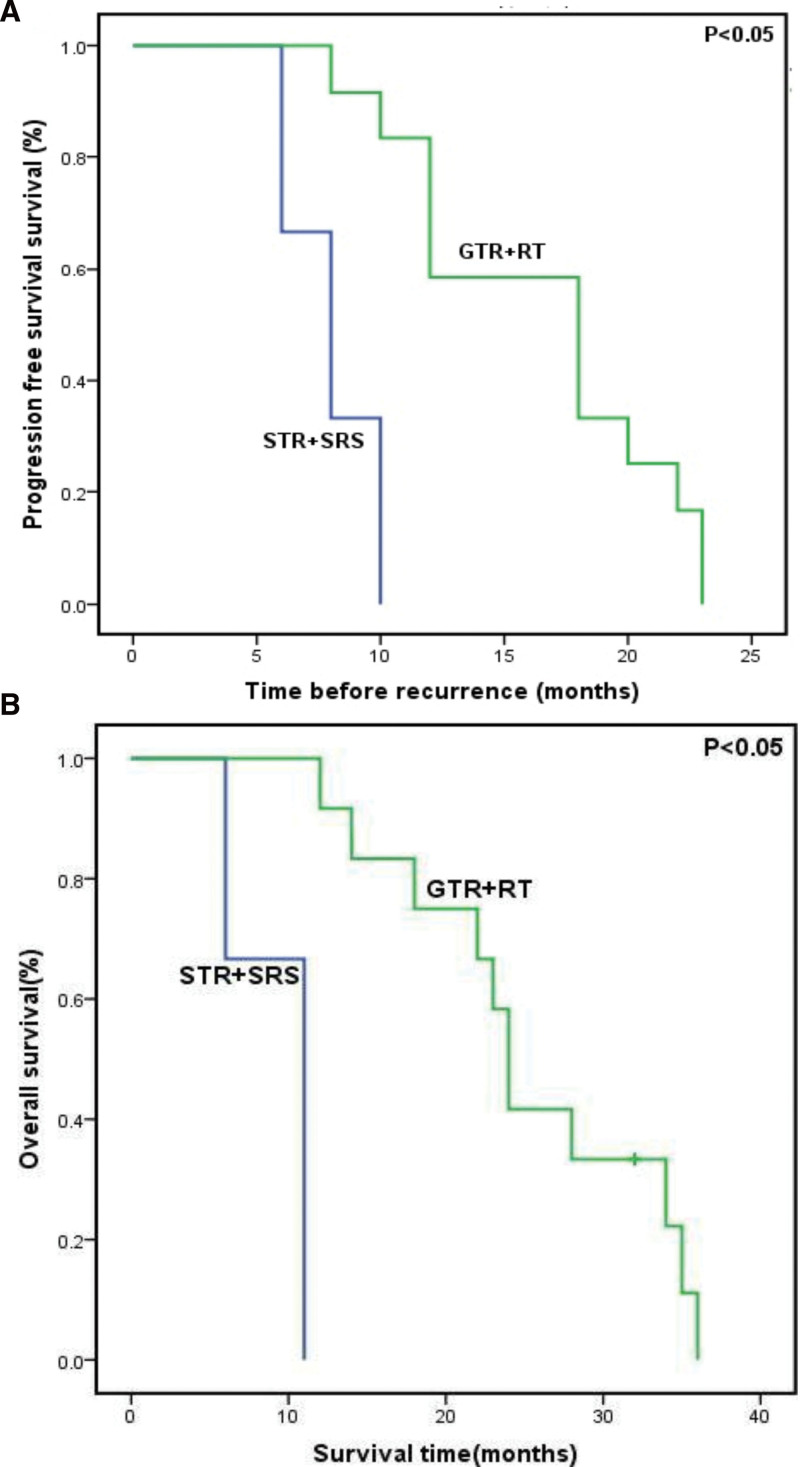
Kaplan–Meier curves comparing the overall survival and progression-free survival of the 2 treatment methods. GTR with RT appeared to extend (A) progression-free survival and (B) overall survival. GTR = gross total resection, RT = radiotherapy, STR = subtotal resection, SRS = stereotactic radiosurgery.

Adjuvant treatments included conventional radiotherapy (RT) and SRS. None of the patients had received adjuvant chemotherapy or biological adjuvant treatment. All 12 patients with GTR underwent postoperative conventional RT with a mean dose of 50 Gy (range, 45–54 Gy) in 1.8 to 2-Gy fractions. Three patients with STR underwent SRS, specifically Cyber Knife radiosurgery (CKRS; Accuray, Inc.). The mean follow-up was 22.6 months (range, 6–36 months). All 15 patients returned to normal life 1 month postoperatively. All 15 patients experienced recurrence or metastasis (Fig. 1H) after an average of 14.7 months (6–23 months): local recurrence (8 cases), distant metastasis (5 cases), and both recurrence and metastasis (2 cases). Of the 15 patients, 5 patients received a second surgery and 1 patient received radiosurgery. The mean overall survival was 22 months (6–36 months; Fig. 5). The median survival time was 23 months (Table 1). Fourteen patients succumbed to a disease associated with the tumor. The overall survival rates at 1, 2, and 3 years were 80%, 47%, and 13%, respectively. The average progression-free survival times were 16 and 8 months in the GTR combined with RT and STR combined with SRS groups, respectively (*P* < .05; Fig. 6A). The average survival times were 25 and 9 months in the GTR combined with RT and STR combined with SRS groups, respectively (*P* < .05; Fig. 6B).

### Pathological examination

A senior neuropathologist verified the results of the histological examination. Microscopic examination revealed a typical histological diagnosis of malignant melanoma (Figs. 1F and 2G). Most tumor cells are rich in melanin and have large nuclei. Giant polygonal or spindle-shaped cells were observed, and mitosis was frequently noted. Immunohistochemical analysis revealed positive reactions for S-100 protein, antihuman melanoma black-45 (HMB-45) antibody, vimentin (VIM), Sox-10, melan-A, vascular endothelial growth factor, and glial fibrillary acidic protein. Positive staining for S-100 protein, HMB-45, VIM, and Sox-10 was observed in 14 cases (93.3%), 13 cases (86.7%), 13 cases (86.7%), and 13 cases (86.7%), respectively. The marker of proliferation, Ki-67, was also examined, and the staining in all the tumors ranged from 10% to 30%.

## 4. Discussion

Intracranial melanomas can be classified as metastatic or primary.^[3]^ PIMMs are a group of rare malignancies of the central nervous system that were first described by Virchow in 1859.^[6]^ The estimated incidence of PIMMs is ~0.5 cases per 10,000,000 person-years^[8]^ and accounts for ~0.13% of all central nervous system tumors in China.^[9]^ The diagnosis of PIMMs should be consistent with that outlined by Willis.^[4]^ In the present report, perioperative examination revealed that none of the patients had non-cerebral melanoma or melanoma in other parts of the body according to their surgical history. All the 15 patients were diagnosed with PIMM. While reports of the association with sex are inconsistent in the literature, a number of studies have reported a male predominance.^[6,8]^ There was a male predominance (male: female, 11:4) in the present study, which is consistent with the literature.^[10]^ The mean age of onset was 37.9 years (19–61 years) in the present study, which is younger than that reported previously.^[1–5]^ This may be due to the small sample size, which is a limitation of this study.

PIMMs can be divided into diffuse meningeal tumors and solitary melanomas.^[3]^ Although PIMMs can occur throughout the central nervous system, they are more likely to develop in the posterior cranial fossa, Meckel cave, and spinal cord, where they typically present with a mass effect.^[6]^ In the present study, 5 cases were located infratentorially, and 1 case was located in the middle and posterior cranial fossa. The clinical symptoms of PIMM are nonspecific and are consistent with those of other malignant brain tumors. the most common clinical symptoms were previously reported to be intracranial hypertension and hydrocephalus (43.2%).^[6]^ In addition, the diffuse type appears to have a higher possibility of intracranial hypertension and hydrocephalus than the solitary type.^[3]^ There were 14 cases of solitary PIMM in the present report. Symptoms at presentation included headaches, vomiting, and focal neurological deficits. The most common clinical symptoms are headache and vomiting due to intracranial hypertension. The mean duration of symptoms was 6.3 months, which is longer than that in common intracranial malignant tumors.

CT and MRI are important techniques used for the preoperative differential diagnosis of PIMMs.^[11–14]^ PIMMs may show high densities on CT scans, which need to be differentiated from hemorrhage.^[15]^ However, some PIMMs also display equal or low densities on CT scans, where they generally lack specificity.^[15]^ MRI scans can reveal typical features of the majority of PIMMs, namely high-signal intensity on T1-weighted images and low-signal intensity on T2-weighted images.^[5,13]^ This is different from other common intracranial tumors, including meningiomas, schwannomas, metastases, and gliomas. There is a bipolar dipole interaction between the unpaired electrons of the stable melanin organic radicals and aquaporin, resulting in the shortening of the T1 and T2 relaxation times and the generation of these typical MRI features.^[15]^ This decrease in relaxation time is directly proportional to melanin content in melanoma.^[14]^

Most tumors with hyperintensity on T1-weighted images can indicate the presence of hemorrhage, fat, or melanin.^[11]^ Furthermore, hypointensity on T2-weighted images may serve as a clue for distinguishing lipomas from melanoma.^[11]^ Therefore, typical T1- and T2-weighted signals may provide clues for the diagnosis of PIMMs. In the present study, 14 tumors were shown to exhibit hyperintensity on T1-weighted images, hypointensity on T2-weighted images, and no or mild enhancement. However, only 3 patients were accurately diagnosed with melanoma before surgery. A definitive diagnosis was not made until a typical black tumor was detected during the operation. Intratumor hemorrhage, which leads to MRI signal confusion coupled with the rarity of melanoma, renders the correct initial diagnosis highly challenging.^[4,13–15]^ However, enhancements in the recognition of the MRI features of melanoma can improve the preoperative diagnosis rate. Therefore, if a lesion shows hyperintensity on T1-weighted images, the possibility of melanoma should be considered.

Pathological examination is the standard protocol for the diagnosis of a wide range of diseases.^[11]^ Malignant melanomas can be diagnosed using routine hematoxylin and eosin and immunohistochemical staining techniques.^[12]^ Malignant melanomas are typically densely pleomorphic, spindle-shaped cells with mitosis, abundant cytoplasm, and rich melanin deposits.^[4]^ In the present study, the tumor tissues were rich in melanin and contained heteromorphous large cells with obvious nucleoli, where giant tumor cells could be seen under a light microscope.

Immunohistochemical staining can be used to differentiate malignant melanomas from other tumors.^[16]^ S-100 is highly expressed (95%) in malignant melanomas, and HMB-45 is another highly expressed and specific pathological marker for diagnosing malignant melanomas.^[6,16]^ VIM is a mesenchymal tumor marker that can provide complementary information for the diagnosis and/or differentiation of PIMMs when combined with other markers.^[6,16]^ In the present study, the positive rates of S-100, HMB-45, and VIM were 93.3%, 86.7%, and 86.7%, respectively, which is consistent with the literature.^[11,12,16]^ Sox-10 has also been shown to be a sensitive marker of cutaneous and uveal melanoma.^[17]^ The positive rate of Sox-10 was 86.7% in the present study.

Numerous previous studies^[1,6,10,17–19]^ appeared to agree that gross total resection is the most effective treatment method for melanoma of the central nervous system. Incomplete tumor removal increases the risk of recurrence and poor prognoses.^[1,6,10,17–19]^ Supporting this, the prognoses of patients who underwent total resection tended to be superior to those of patients who underwent incomplete resection.^[3,19]^ Total resection combined with postoperative RT seems to be the preferred treatment for eliminating mass effects, improving preoperative symptoms, and achieving histological diagnosis.^[3]^ Rodriguez et al^[10]^ reported that the mean survival in patients who underwent total tumor resection (19.6 months) was significantly longer than that in patients who underwent partial resection or biopsy (9.3 months). In addition, Man and Wang^[8]^ previously reported that complete surgical resection could increase the survival rate, whilst the age of <19 years and intracranial tumor were independent factors of poor prognosis. However, STR or biopsy combined with RT or chemotherapy cannot improve patient survival of patients.^[6,16,20]^ The present study further validates the results of the present study, whereby GTR with RT appeared to extend progression-free survival. The average overall survival time of the GTR group was significantly higher than that of the STR group. Surgical resection was performed based on the patient’s symptoms and location, size, and number of lesions. Total resection should be attempted using microsurgical techniques to protect the nerve function. However, it is difficult to achieve total resection due to the occult onset of the tumor, abundant blood supply to the tumor, and close proximity of the tumor to important neurological structures. In this group of patients, GTR was achieved in 80% cases, whereas STR was achieved in 20% cases because the tumors were in close contact with the pyramidal tract.

Yamane et al^[21]^ previously reported the mean survival of patients with solitary tumors to be 20.7 months. In addition, Man and Wang^[8]^ reported that the 1-year, 2-year, 3-year, and 5-year survival rates of primary melanoma in the central nervous system were 89.3%, 75.6%, 65.2%, and 37.7%, respectively, with a median survival rate of 15 months. In the present study, the median survival time was 23 months for 15 patients. Therefore, the treatment effect in the present study was deemed consistent with that reported in the literature. However, owing to the small number of cases, it is necessary to further increase the number of cases to verify the treatment outcome.

Although melanoma has been frequently reported to be insensitive to the commonly used doses of RT, various studies have also found that the addition of adjuvant RT following surgery can significantly reduce the risk of local recurrence compared to resection alone.^[19,22–25]^ In particular, the combined application of WBRT and SRS was found to be more effective compared with that of either WBRT or SRS alone.^[19,23]^ Furthermore, the prognosis of patients undergoing microsurgery combined with SRS and/or WBRT was observed to be superior compared with that of patients who underwent either microsurgery or WBRT alone.^[19,23]^ In the present study, 12 patients with GTR received adjuvant RT postoperatively, whereas the other 3 patients with STR received CKRS. The average overall survival time of the GTR combined with RT group was significantly higher than that of the STR combined with CKRS group.

However, the effects of adjuvant chemotherapy on PIMMs remain controversial. There is little evidence that chemotherapy can significantly affect PIMMs.^[19]^ Chemotherapeutic drugs generally lack efficacy in cerebral tumors because they cannot penetrate the blood–brain barrier.^[19]^ It has been previously reported that when tumors grow within the brain parenchyma, the blood–brain barrier structure and function can become damaged, increasing permeability.^[19]^ Adjuvant chemotherapy has been observed to have limited effects on the management of metastatic melanoma.^[19]^ However, none of the 15 patients in the present study received postoperative chemotherapy. Several studies have^[19,26–30]^ reported that immunotherapy can potentially prolong the overall survival of patients with metastatic melanoma. Programmed cell death protein 1 inhibitors, such as pembrolizumab, were found to prolong the progression-free and overall survival of patients with advanced melanoma.^[31]^ In addition, the prospect of gene therapy (targeted therapy) for PIMMs has been previously explored.^[19,32,33]^ Gene therapy (targeted therapy) combined with immunotherapy may improve the prognosis of patients with metastatic melanoma compared with immunotherapy combined with RT.^[34]^ However, all of the aforementioned findings require further evaluation. The prognosis of patients with PIMM who undergo radical resection combined with postoperative RT remains poor. There have been a number of reports on PIMM regarding the effects of immunotherapy or targeted therapy^.[19,32–35]^ Inhibition of melanogenesis in advanced melanotic melanoma represents a realistic adjuvant strategy to enhance the efficacy of immunotherapy, radiotherapy, and chemotherapy.^[35]^ These strategies may open a new era of PIMM treatment.

A solitary tumor with leptomeningeal enhancement may have similar features to diffuse leptomeningeal melanosis, which is considered to be benign but has an exceptionally poor prognosis with a mean survival of 6.7 months.^[1]^ Patients with diffuse leptomeningeal enhancement who had no surgical indications were excluded. Additional patients and studies are required to confirm the role and efficacy of surgery and adjuvant therapy in PIMM. In addition, the incidence rate of this tumor is low, the majority of which are reported in the form of case reports in the literature. Therefore, it is difficult to compare the characteristics reported in the present study with those reported in previous studies.

## 5. Conclusion

Data from the present study suggest that PIMM is a rare malignancy with poor prognosis. However, radical resection with RT may result in higher overall survival rates. Targeted immunotherapy may be a promising treatment option for PIMM.

## Author contributions

**Data curation:** Yang Yang, Dongmei Li.

**Formal analysis:** Dongmei Li.

**Investigation:** Lifeng Chen.

**Methodology:** Lifeng Chen.

**Project administration:** Lifeng Chen.

**Writing – original draft:** Lifeng Chen.

**Writing – review & editing:** Lifeng Chen, Bo Bu.
